# Baltimore Academy of Medicine

**Published:** 1881-06

**Authors:** 


### BALTIMORE ACADEMY OF MEDICINE.

*Blindness Followed by Ascending Progressive and Fatal Paralysis.*—Dr. J. J. Chisolm reported the case of a gentleman, aet. 40, and weighing 180 to 200 pounds, who one day (having previously had no symptom of any eye or other trouble) found his eyes painful on motion, after having used them for one or two hours. The sight of one eye, impaired during the day, was lost by night. The next day a similar occurrence took place in the other eye, and the patient became in a few hours totally blind. After forty-eight hours, receiving no benefit from anything done for him, he came to Baltimore. On arriving at Dr. Chisolm's office, he was noticed to have some difficulty in walking; the pupils were found to be widely dilated, the opthalmoscope revealed choked disks with effusion in both. There was nothing else discoverable in the case. He was leeched, and iodide of potassium given in large doses. The next morning he had lost the power of feeling in either foot, also the control of the sphincter ani muscle and the ability to pass his urine. He felt tickling in his legs. The following day he lost sensation and the power of moving his lower limbs from his hips downwards The paralysis crept slowly on upwards, until the abdominal muscles became involved, and respiration had to be carried on by the thoracic muscles alone. Finally the diaphragm alone acted in respiration He died in six days, his mind being clear to the last. No autopsy was permitted.

The case was curious, in that the trouble began with the vision, and then darted from the other extremity, in the other senses being good, whilst vision alone was lost, and then of sensation, and then there was loss of motion. There was no evidence of syphilis in the case, and the patient had been perfectly well up to the attack.

*Unusual Situation of Diphtheritic Membrane.*—Dr. Chisolm reported two cases of diptheritic deposit confined exclusively to the lower eye-lids. The glands at the angle of the jaw were enlarged. There was no febrile excitement. Iodoform locally applied caused rapid disappearance of the membrane.

Dr. Arnold mentioned the case of a child who, three days after the operation of circumsion, was taken ill. A large diphtheritic patch formed on the wound. The cervical glands were enlarged, though nothing was to be seen on examining the throat. The glands in the groins were not enlarged. The child gradually sank and died. Diphtheria was present in the neighborhood at the time.

*Divergent Squint.*—Dr. Theobald reported a case of divergent squint in a lady twenty-five years of age, the result of an operation for convergent squint performed fifteen years previously, in which he obtained an excellent result from advancement of the tendon of the internal rectus muscle of the squinting eye, combined with tenotomy of both external recti muscles. The method of securing the tendon in its advanced position, suggested by Brudenell Carter, in which the needles are passed under the conjunctiva and brought out over the insertion of the superior and inferior recti muscles respectively, so as to include a broad conjunctival bridge, was adopted. The effect of the operation was not only satisfactory from a cosmetic point of view, but still more important, the restoration of binocular vision was secured.

*Opium Poisoning in an Infant Successfully Treated by Belladonna.*—Dr. Chew reported the case of a child, aged three months, who was given by mistake a powder containing one-fourth grain of morphia. The mistake was not suspected until six-and-a half hours after, when Dr. Chew was called in. The little patient was then in a deep coma, which was becoming more and more profound. Tincture of belladonna was administered at short intervals, in one-and-a-half drop doses and shaking; coffee and the application of ice to the spine were at the same time employed. In one hour and-a-half atropinism was produced, and simultaneously improvement in the child's condition was manifest. In four-and-a-half hours after the doctor first saw it, it was considered out of danger and allowed to go to sleep.

*Intra-Uterine Fibroid Tumor.*—Dr. H. P. C. Wilson exhibited a specimen of fibroid tumor removed this afternoon from a patient, aet. 38 She had had hemorrhages for five or six years, being well only six or eight days in each month. In consequence, she was greatly debilitated and very anæmic, and at times suffered great pain. She was unmarried and her cervix was small, indurated and conical, admitting a probe with difficulty into the uterine cavity. It seemed impossible to get into the uterus sufficiently to remove the tumor, and oophorectomy seemed the only resource left to save her life. By the use of dilators and sea-tangle tents of gradually increasing size (first immersed in glycerine and carbolic acid), he secured sufficient dilation to enable him to discover a tumor occupying the uterine cavity. The os internum was next split both in front and behind, and when the patient had recovered from this operation and from menstruation, which had meanwhile come on, the use of the tents and dilators was resumed. Yesterday the cervix was packed with as many sea tangle tents as could be gotten into it, and on removal to-day, a Barnes' dialator was introduced, filled with hot water and left in *situ* about one hour, when—the patient being under chloroform—a volsellum forceps was passed in and the tumor seized and drawn down.

It was found to be sessile and attached to the fundus. The uterus being steadied by a hand on the abdomen, the tumor was sawed away in several sections with Thomas' scoop; a portion was also cut away with the saw scissors, the whole of it thus being removed. Five-and-a half weeks were required to prepare the patient for the operation, which consumed two-and-a-fourth hours. The operation was completed by passing a small syringe to the fundus, washing out the cavity gently with warm water and carbolic acid, and then mopping out the uterus with Monsel's solution—carbolic acid and glycerine. Her prospects at the present time are favorable. (Nearly four months have elapsed since the case was reported, and Dr. Wilson reports the patient as perfectly well and menstruating normally.—REPORTER.)

Dr. McKew inquired whether a fusiform cervix with so large a body, was not rare.

Dr. Wilson replied, No. The cervix would have been obliterated had the tumor been allowed to remain and grow so as to bring its dilating force to bear upon the internal os and cervical canal. It depends much upon the seat of attachment of the tumor; if it be attached at the fundus, non-dilation of the cervix is not uncommon. Dr. Wilson referred to a patient with a fibroid in the uterus—whether interstitial or not, he was uncertain—in which the os was unaffected. The patient has had pelvic cellulitis and peritonitis, and, in consequence, the doctor was afraid to introduce a tent in order to explore the uterine cavity. In another case, with a large fibroid in the walls of the uterus, the os could not be dilated by the use of tents. There are as many cases of this sort without dilation of the cervix as with it.

*Paralyzation of Heart in Diphtheria.*—Dr. Chew reported a case of a girl, aet. 7, who had a slight redness of the throat—apparently a simple pharyngitis. The next day a diptheritic membrane was visible. The case soon developed into the gangrenous form, and large gangrenous masses came away from the faucial and nasal surfaces. Carbolic acid spray was employed and sublimed sulphur was thrown over the entire diseased part. The patient did well for ten days; then a sudden change took place; the pulse fell to 44, and there was a manifest tendency to cardiac paralysis. There was some difficulty in swallowing, followed by regurgitation of food. For several days, life had to be sustained by injections of brandy and milk. She revived and the pulse went up to 52. Again the heart gave way, and the pulse fell again to 44. A large syringe-ful of brandy was then injected hypodermically into the arm, after which the stomach bore stimulants, and the pulse rose to 80, though still feeble. The urine, once and awhile, was loaded with albumen. The danger in such cases is from sudden paralyzation of the heart. Jenner reports a pulse of 16 per minute. Dr. Chew insisted on the importance of injecting the nostrils in nasal diphtheria, as urged by Jacobi.

Dr. McKew reported the case of a child, aet. 5, who had an attack of faucial diphtheria, accompanied with extreme feebleness. She was convalescing from this, and was found one day at noon playing with her dolls, lying on her couch and apparently out of danger. Shortly after this visit, she arose suddenly to her feet to get a doll which had fallen upon the floor, when she fell back and was found to have expired.

*Extra Uterine Pregnancy—Removal of Decomposing Fœtus Through Umbilicus.*—Dr. Erich exhibited a specimen of a decomposing female fœtus of about nine months development with the following history: In February, 1880, the patient noticed that she was getting "a lump in her stomach." Towards the latter part of the following spring she quickened. She continued to grow larger until August; then the swelling began to diminish, and violent cramp-like pains with symptoms of peritonitis appeared. In October, pus began to discharge through the umbilicus. Two days ago (*i. e.*, April 3rd, 1881,) the patient came under care. She emitted a most offensive odor. The head of one femur projected from the umbilicus. To-day the entire fœtal remains were removed. No cutting was done, and as little interference with the sac as possible was practised for fear of making an opening through some other portion of the sac weakened by the degenerative process. A portion of the scalp was found attached to the cyst wall, and was removed. The parietal and frontal bones were found lying loose in the cyst. Evidently the fœtus had reached term early in August; the liquor amnii had then been absorbed, and in consequence of this peritonitis had been set up. There was no sign of the placenta, which probably was absorbed. The after treatment consisted in filling the cyst with carbolized cotton, so as to cause it to heal from the bottom, and cause all the decomposing matter to be thrown off. The patient is doing well.

Dr. McKew could not suppose the placenta to have been absorbed without more serious symptoms.

Dr. Taneyhill suggested that the greater part of the placenta may have been discharged in the decomposing fluids.

Dr. B. B Browne said in most of these cases the placenta is attached to the Fallopian tube or some of the adjacent tissues; in Dr. Wilson's case of "Extra-Uterine Twin Fœtation," it was attached to the broad ligament. In this event it may remain entirely or partially unabsorbed and give no trouble.

*Complete Inversion of Uterus During Labor.*—This case, reported by Dr. Reiche, occurred in a patient, aet. 28, married in 1875, and who had had three consecutive breech presentations and one miscarriage at two months, there being retention of the placenta of three of these. The fifth pregnancy had advanced to about eight-and a-half months, when, on August 13, 1880, labor took place—the breech presenting. After some delay, owing to the want of pains, the child was expelled, apparently dead, but was resuscitated. A tumor was found projecting through the os uteri, supposed to be the placenta. The pains had been entirely arrested since the beginning of the expulsive period, which had been completed by the voluntary efforts of the woman alone. Suddenly a sharp pain appeared, the patient placed her hands on her loins, and with a violent effort, the uterus was forced out between her thighs. The placenta was adherent and so firmly, that it was with difficulty detached, and then only in small masses at a time. The uterus was changed in shape—that part occupied by the placenta being soft, projecting and *completely flaccid.* The patient complained of excruciating pain in the left leg. An effort was made to restore the organ to its place, which was arrested by the collapse and cessation of breathing of the patient. Hæmorrhage, which was profuse, was relieved only by sponging the placental site with Monsel's solution and glycerine. Dr. Erich was called in consultation, with whose assistance, under chloroform, restoration was effected—that part of the organ inverted last, being first returned. Fluid extract of ergot (ʒij) was then injected deep into the abdominal muscles. Fever ensued with tympanites and nausea. There was a fœtid lochial discharge, the offensive odor of which was relieved by boracic acid suppositories. The paralysis of the uterus continued until August 27th, when the whole organ was found to be well contracted. There was no negligence in this case, no mechanical or exciting cause to which it could be referred. It was the result of paralysis of the placental site, clearly made out, and lasting for some time after reposition.

Dr. Erich said the best means of dilating the cervix was by introducing one finger and then adding others until sufficient space was obtained for the return of the inverted position. In the present case, the placental site of the uterus was not only paralyzed, but thinner than the rest of the organ. Boraric acid, as employed, has decided advantages over carbolic acid, being perfectly harmless (which is far from being the case with carbolic acid), and being but slightly soluble in water, remains in the vagina for several hours. Most cases of inversion depend upon paralysis of the placental site. Traction upon the cord is extremely common, every old midwife practicing it; yet how rare is inversion. Dr. Erich looked upon hour-glass contraction as due to this same form of paralysis. He also related a case in which the bulging of the placental site was mistaken by the physician for a subserous fibroid.

Dr. Morris had had two cases of inversion of uterus during labor in his experience, in both of which restoration was effected without difficulty.

